# Impact of Bone Grafting and Graft Type on Fusion and Patient‐Reported Outcomes Following Subtalar Arthrodesis: A Multicenter Retrospective Cohort Study

**DOI:** 10.1002/jfa2.70160

**Published:** 2026-05-12

**Authors:** Giovan Giuseppe Mazzella, Antonio Bove, Andrea De Fazio, Natale Maria Gangemi, Marco Peruzzi, Fabrizio Forconi, Philip Krekosch, Christian Lausmann, Mustafa Citak, Vincenzo Di Sanzo, Giulio Maccauro, Raffaele Vitiello

**Affiliations:** ^1^ Department of Aging, Orthopaedic and Rheumatological Sciences Fondazione Policlinico Universitario Agostino Gemelli IRCCS Rome Italy; ^2^ Madonna delle Grazie Clinic Velletri Rome Italy; ^3^ Villa Stuart Sport Clinic FIFA Medical Center of Excellence Rome Italy; ^4^ Department of Orthopaedic Surgery Helios ENDO—Klinik Hamburg Hamburg Germany

## Abstract

**Background:**

The role of bone grafting in subtalar joint arthrodesis (SJA) remains controversial. This study aimed to compare clinical and radiographic outcomes of SJA performed with and without bone graft and to evaluate the influence of different graft types on fusion and functional results.

**Methods:**

A multicenter retrospective observational study was conducted including 66 patients who underwent isolated SJA between 2023 and 2025. Patients were divided into graft (*n* = 51) and no graft (*n* = 15) groups. A subgroup analysis compared autologous, fresh frozen allogeneic, and commercial allogeneic grafts. Outcomes included osseous union, time to union, complications, and functional scores (AOFAS, FAAM‐ADL, and FAAM‐Sports). Multivariable regression and ROC analyses were performed to identify independent predictors of nonunion and delayed union.

**Results:**

Overall, the union rate was 90.9%. Union was achieved in 92.2% of grafted patients and 86.7% of nongrafted patients (*p* = 0.612). Bone graft use was not independently associated with union, complications, or time to union in the adjusted exploratory analyses. Increasing age and BMI were independently associated with a prolonged time to union. ROC analysis identified age ≥ 60 years as a predictor of nonunion (AUC 0.782) and age ≥ 59 years and BMI ≥ 25.9 kg/m^2^ as predictors of delayed union. Both groups showed significant postoperative improvements in all functional scores (all *p* < 0.001). Autologous graft was associated with higher postoperative functional scores, although this finding should be interpreted cautiously given potential baseline differences and selection bias.

**Conclusions:**

In isolated SJA performed in a well‐aligned hindfoot, high union rates and significant functional improvement were achieved regardless of bone graft use. However, due to the retrospective, nonrandomized design and limited number of nonunion events, no definitive conclusions can be drawn regarding the routine necessity or superiority of bone grafting. Though, bone graft use appeared to be associated with improved functional outcomes in selected higher risk patients, although these findings should be interpreted cautiously given the exploratory nature of the analysis. All subgroup and threshold analyses should be interpreted as exploratory.

## 1 Introduction

### 1.1 Subtalar Joint Biomechanics and Osteoarthritis

The subtalar joint (SJ) plays a key role in complex kinematics of the hindfoot during gait [1]. Its motion is triplanar: inversion, adduction, and plantarflexion occur simultaneously, resulting in a ‘down and in’ movement, whereas eversion, abduction, and dorsiflexion combine to generate an ‘up and out’ motion [2]. Subtalar joint osteoarthritis (SJ OA) is characterized by progressive degeneration of the articular cartilage of the talocalcaneal joint, leading to pain, stiffness, loss of hindfoot motion, and impaired gait mechanics.

### 1.2 Clinical Presentation and Epidemiology

Unlike ankle osteoarthritis, which is frequently post‐traumatic, SJ OA may develop as a consequence of both acute trauma and chronic biomechanical alterations affecting hindfoot alignment and load distribution. Clinically, patients often report deep hindfoot pain exacerbated by uneven terrain, limited tolerance to prolonged walking, and difficulty with activities requiring hindfoot adaptability. Epidemiologically, SJ OA is less common than tibiotalar arthritis but represents a clinically significant source of disability. Its true prevalence is difficult to quantify, as isolated SJ OA is frequently underdiagnosed or associated with concomitant hindfoot or ankle pathology [3]. Available data suggest that post‐traumatic arthritis following calcaneal fractures accounts for up to 60%–80% of cases, whereas primary degenerative disease and inflammatory arthropathies represent less frequent etiologies [4, 5]. SJ OA is most commonly diagnosed in middle‐aged and older adults, with incidence increasing after the fifth decade of life, particularly in patients with a history of hindfoot trauma. Subtalar joint arthrodesis (SJA) is widely accepted as a surgical treatment for degenerative disease of the SJ when conservative measures have proven unsuccessful. Indications include hindfoot disorders secondary to post‐traumatic, degenerative, or rheumatoid arthritis, neuromuscular conditions, talocalcaneal coalition, and structural hindfoot deformities [6, 7]. Prior to surgical intervention, conservative treatment options are commonly employed and may include activity modification, nonsteroidal anti‐inflammatory drugs, orthotic devices aimed at hindfoot stabilization, physical therapy, and intra‐articular corticosteroid injections. Although these measures can provide temporary symptom relief, their efficacy is often limited in advanced disease, and many patients ultimately require surgical management due to persistent pain and functional limitation.

### 1.3 Surgical Indications and Risk Factors for Nonunion

The rationale for SJA is to achieve pain relief and restore physiological hindfoot alignment, with the ultimate goal of improving mobility. Symptom reduction is obtained through stable bony fusion, which abolishes abnormal shear at the joint, while realignment of the hindfoot helps normalize load distribution across the articulation [8]. Several patient‐related and disease‐specific factors have been identified as risk factors for the development and progression of subtalar joint osteoarthritis, including prior calcaneal fracture, hindfoot malalignment, obesity, inflammatory arthropathies, smoking, and neuromuscular disorders [9]. In particular, residual deformity after calcaneal fracture fixation and chronic valgus or varus hindfoot alignment have been shown to accelerate degenerative changes within the subtalar joint. Compared with triple arthrodesis, isolated SJA preserves approximately 50% of the residual midtarsal range of motion [10, 11]. Nevertheless, significant postoperative complications such as nonunion and malunion have been reported [12]. Nonunion remains one of the most challenging complications following SJA, with reported rates ranging from 5% to over 20% in the literature. Multiple risk factors for nonunion have been described, including smoking, diabetes mellitus, peripheral vascular disease, obesity, poor bone quality, post‐traumatic etiology, previous hindfoot surgery, and inadequate fixation or joint preparation [13, 14]. Additionally, compromised local biology and altered vascularity following calcaneal fractures may further predispose patients to impaired fusion [15].

### 1.4 Role of Bone Grafting and Study Rationale

The role of bone grafting in SJA remains controversial. Ongoing debate concerns its potential to reduce the risk of nonunion and to influence postoperative outcomes. Autologous bone grafts, allografts, and bone graft substitutes have all been proposed as adjuncts to enhance fusion by improving the biological environment at the arthrodesis site [16]. Although some authors advocate routine graft augmentation, particularly in high‐risk patients, others report comparable fusion rates without grafting when meticulous joint preparation and stable fixation are achieved [17]. Accordingly, the present study was designed to explore associations between bone graft use and the graft type with fusion outcomes and patient‐reported outcome measures in isolated SJA, without the intent to establish causal relationships. In addition, this study provides incremental value by focusing specifically on isolated subtalar arthrodesis in a well‐aligned hindfoot, reflecting a real‐world multicenter setting and integrating both radiographic and patient‐reported outcomes in relation to graft selection.

## 2 Materials and Methods

### 2.1 Study Design and Participants

This was a multicenter retrospective observational study based on the analysis of routinely collected clinical data. Participants from multiple centers were included in the study in order to increase the sample size and improve the external validity of the findings. The study was conducted in accordance with established methodological recommendations for observational studies.

All consecutive patients who underwent subtalar arthrodesis and met the predefined inclusion criteria during the study period from July 1st, 2023, to July 1st, 2025 were eligible for inclusion.

Inclusion criteria were as follows:

— Age > 18 years,
— Diagnosis of subtalar joint pathology requiring arthrodesis, including primary osteoarthritis, post‐traumatic osteoarthritis, or inflammatory/immune‐mediated disease,
— Availability of clinical and radiographic follow‐up sufficient for outcome assessment, and
— Patients who provided informed consent to participate in the study.

Exclusion criteria were as follows:

— Previous hindfoot arthrodesis involving adjacent joints,
— Hindfoot malalignment,
— Active infection at the time of surgery, and
— Incomplete clinical or radiographic data.

The decision to use bone graft and the choice of the graft type were based on surgeon preference and intraoperative assessment rather than on predefined criteria. As such, this study was not designed to support causal inference, and all comparative analyses should be interpreted as exploratory and hypothesis‐generating.

**FIGURE 1:**
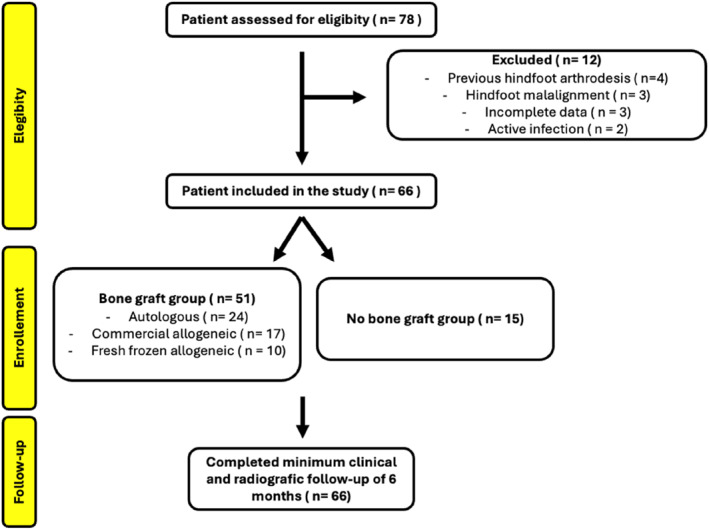
STROBE flow diagram illustrating patient eligibility, exclusions with reasons, allocation according to bone graft use, subgroup analysis by the graft type, and inclusion in the final analysis.

Patient selection and inclusion are reported according to the STROBE guidelines for observational studies. A flow diagram detailing the number of eligible patients, exclusions, and final study population is provided in Figure 1.

### 2.2 Data Collection

Clinical data were retrospectively extracted from electronic medical records and institutional databases of the participating centers. Collected data included

— Demographic characteristics (age, sex, BMI, and comorbidities),
— Underlying pathology leading to subtalar arthrodesis,
— Surgical details, including the use and type of bone graft,
— Imaging and intraoperative findings, when applicable, and
— Primary and secondary outcome measures.

A standardized data collection form was used across all centers to ensure data consistency and homogeneity.

### 2.3 Study Outcomes

Clinical outcomes were assessed using validated patient‐reported outcome measures, including

— The American Orthopaedic Foot and Ankle Society (AOFAS) hindfoot score,
— The Foot and Ankle Ability Measure‐Activities of Daily Living (FAAM‐ADL);
— The Foot and Ankle Ability Measure‐Sports (FAAM‐Sports).

Donor‐site pain was assessed at 1 month, 6 months, and 12 months postoperatively using the visual analog scale (VAS) in patients who underwent autologous bone graft harvesting, in order to assess donor‐site morbidity.

*Bone union was assessed using lateral, oblique, and hindfoot axial radiographs*. Successful bone union was defined as clinical resolution of hindfoot pain with radiographic evidence of osseous trabecular bridging involving more than 50% of the posterior facet surface and no evidence of screw loosening during follow‐up, typically within the first 6 months after surgery [18, 19]. Fusion assessment based on standard radiographs may be less sensitive than CT in detecting partial union. The > 50% trabecular bridging criterion, although commonly used, is inherently subjective when applied to plain radiographs and may introduce misclassification bias. Radiographic follow‐up was not strictly standardized across centers. Although imaging was routinely performed at approximately 6 weeks, 3 months, and 6 months postoperatively, the exact timing and frequency of radiographic assessments varied according to local clinical practice. As a result, time‐to‐union analyses may partially reflect follow‐up scheduling in addition to biological healing.

Patients were primarily divided into two groups based on the use of bone graft (graft vs. no graft). A secondary subgroup analysis was conducted within the graft group to compare outcomes among different graft types, namely autologous bone graft, fresh frozen allogeneic (heterologous) bone graft, and commercially available allogeneic bone graft.

### 2.4 Surgical Technique

After induction of general or spinal anesthesia, the patient was placed in a semilateral position with the affected limb uppermost. A pneumatic tourniquet was applied to the mid‐thigh. A lateral sinus tarsi approach to the subtalar joint was performed. A longitudinal skin incision was made approximately 1 cm posterior and inferior to the border of the lateral malleolus. The sural nerve and peroneal tendons were carefully identified, dissected, and protected throughout the procedure. Following incision of the calcaneal periosteum, the subtalar joint was exposed. Residual articular cartilage, osteophytes, and fibrous tissue surrounding the joint were meticulously removed. The articular surfaces were prepared with an osteotome and a curette until bone bleeding was observed, confirming adequate preparation for fusion. When indicated, bone grafting was performed using autologous, fresh frozen allogeneic, or commercially allogenic bone graft, according to surgeon preference and local availability. Autologous cancellous bone graft, harvested from the ipsilateral iliac crest (or local morselized bone when appropriate), was packed into the subtalar joint space to facilitate osteogenesis. Fresh frozen allogeneic (heterologous) bone graft consisted of cancellous bone obtained from femoral heads provided by a hospital or an accredited bone bank. Commercial allogeneic bone graft (MAXGRAFT, Botiss biomaterials GmbH, Zossen, Germany) was obtained from human donor bone, processed by the manufacturer to remove immunogenic components while preserving the native bone matrix and its osteoconductive characteristics, and used according to the manufacturer's instructions. The volume and the size of the graft were individually selected by the operating surgeon based on the intraoperative assessment of residual gaps and the morphology of the subtalar fusion surfaces. Although no formal algorithm was adopted, bone grafting was generally favored in the presence of residual joint gaps, reduced bone bleeding after joint preparation, sclerotic subchondral bone, or compromised bone stock. These criteria were applied pragmatically across centers. The subtalar joint was then reduced in a neutral to slight valgus position, ensuring appropriate hindfoot alignment. Once adequate bony apposition and proper hindfoot alignment were achieved, temporary fixation was obtained using K‐wires inserted from the calcaneal tuberosity into the talar dome across the posterior facet. Correct positioning was confirmed under fluoroscopic guidance. Definitive fixation was then performed using two 6.5 mm screws, consistent with the current gold standard for subtalar joint arthrodesis. All screws were partially threaded cancellous screws. Proper reduction, screw placement, and alignment were verified on anteroposterior and lateral fluoroscopic images. After thorough irrigation with normal saline, hemostasis was secured. Layered wound closure was performed using absorbable sutures for the deep layers and nonabsorbable sutures for the skin. A sterile dressing and a below‐knee posterior splint were applied with the ankle maintained in a neutral position. Although a standardized surgical approach was adopted across centers, minor variations in surgical technique, fixation strategy, and graft handling were present according to surgeon preference. However, the core principles of joint preparation, alignment, and fixation were consistent.

### 2.5 Postoperative Management and Follow‐Up

Postoperatively, all patients followed a comparable rehabilitation protocol across centers. The operated limb was immobilized in a below‐knee splint or cast for the first 2 weeks. Sutures were removed at approximately 14 days, followed by transition to a removable walker boot. No weight‐bearing was maintained for the first 6 weeks, after which progressive partial weight‐bearing was allowed based on clinical and radiographic assessment. Full weight‐bearing was generally permitted between 10 and 12 weeks postoperatively, depending on pain, stability, and radiographic signs of progression toward fusion.

### 2.6 Statistical Analysis

All statistical analyses were performed using Python (SciPy and Statsmodels). Continuous variables were summarized as mean ± standard deviation (SD), while categorical variables were reported as absolute numbers and percentages. Data distribution was assessed using the Shapiro–Wilk test to guide the selection of appropriate statistical tests.

For between‐group comparisons of continuous variables, Welch's *t*‐test was used when normality assumptions were not rejected; otherwise, the Mann–Whitney *U* test was applied. Categorical variables were analyzed using Fisher's exact test for dichotomous outcomes and the chi‐square test for variables with more than two categories.

Preoperative and postoperative functional outcomes (AOFAS, FAAM‐ADL, and FAAM‐Sports) were compared within groups using paired t‐tests when the distribution of within‐subject differences was approximately normal (Shapiro–Wilk *p* > 0.05) and Wilcoxon signed‐rank tests otherwise.

Multivariable analyses were prespecified to adjust for clinically relevant covariates, including age, sex, body mass index (BMI), and graft exposure. For binary endpoints (osseous union and postoperative complications), bias‐reduced penalized logistic regression with Firth correction was employed to mitigate small‐sample and rare event bias, as well as potential data separation. Results are reported as adjusted odds ratios (ORs) with 95% confidence intervals (CIs) and two‐sided *p*‐values.

Time to union was analyzed using linear regression with heteroskedasticity‐consistent robust standard errors (HC3). Given the nonnormal distribution of this variable, time to union was log‐transformed, and results are presented as exp(*β*) (time ratios), corresponding to the relative percentage change in time to union. Accordingly, the estimated associations for time to union should be interpreted as relative differences in healing duration rather than as formal hazard‐based estimates.

Postoperative functional outcomes were further analyzed using ANCOVA‐style multivariable linear regression models, adjusting for the corresponding baseline (preoperative) score in addition to age, sex, BMI, and graft exposure. Regression coefficients are reported as *β* values (points) with 95% CIs and robust (HC3) *p*‐values. A multivariable analysis was performed within the high‐risk subgroup, defined as patients aged > 59 years and/or with BMI > 25.9 kg/m^2^. The postoperative AOFAS score was modelled using an ANCOVA‐style multivariable linear regression including the preoperative AOFAS score, bone graft use (YES vs. NO), age (years), and BMI (kg/m^2^). To reduce sensitivity to heteroskedasticity in this smaller subgroup, heteroskedasticity‐consistent robust standard errors (HC3) were used.

When comparing the three bone graft categories (autologous, fresh frozen allogeneic, and commercial allogeneic), the graft type was entered into the models as a categorical variable using dummy coding, with autologous graft as the reference category. Direct comparisons between commercial allogeneic and fresh frozen allogenic grafts were obtained through post‐estimation Wald contrasts.

Receiver operating characteristic (ROC) analyses were performed to identify clinically relevant thresholds using Youden's J index. Specifically, ROC curves were used to evaluate age in relation to nonunion and age and BMI in relation to delayed union. Delayed union was defined as a time to union greater than 12 weeks, generating a binary outcome. For each ROC analysis, the area under the curve (AUC), sensitivity, and specificity were reported.

Given the limited number of nonunion events, multivariable regression models and ROC analyses are subject to instability and potential overfitting. Therefore, all multivariable, subgroup, and threshold analyses were prespecified as exploratory and hypothesis‐generating, and no formal adjustment for multiple comparisons was applied.

Statistical significance was set at *p* < 0.05 (two‐sided). All regression models were fitted using complete case analysis for the variables included in each model, with the corresponding denominators reported for each analysis.

### 2.7 Ethical Considerations

The study was conducted in accordance with the principles of the Declaration of Helsinki. Due to the retrospective design, informed consent was waived or obtained according to local institutional regulations. Ethical approval was obtained from the institutional review board or ethics committee of each participating center prior to data collection.

## 3 Results

### 3.1 Baseline Demographic and Clinical Characteristics

A total of 66 patients who underwent subtalar arthrodesis were included in the study. The cohort consisted of 28 females (42.4%) and 38 males (57.6%), with a mean age of 53.7 ± 14.2 years and a mean body mass index (BMI) of 25.7 ± 3.2 kg/m^2^.

Regarding comorbidities, 14 patients (21.2%) were affected by rheumatoid arthritis, 22 patients (33.3%) had hypertension, 12 patients (18.2%) had diabetes mellitus, 4 patients (6.1%) showed a history of myocardial infarction, and 6 patients (9.1%) had chronic kidney disease.

Baseline health–related quality of life scores showed a mean PCS‐12 of 36.7 ± 3.4 and a mean MCS‐12 of 48.1 ± 2.0.

The mean operative time for subtalar arthrodesis was 76.1 ± 24.2 min. The mean clinical follow‐up duration was 14.5 ± 7.6 months. All patients had a minimum clinical and radiographic follow‐up of 6 months. Although a minimum follow‐up of 6 months was required for inclusion, most unions were achieved within the first 12 weeks, and radiographic assessment beyond this time point was primarily aimed at confirming consolidation and excluding delayed complications.

Among patients who received autologous bone graft (*n* = 24), the mean donor‐site VAS pain score was 5.83 ± 1.05 at 1 month postoperatively and showed a progressive reduction over time, reaching 2.0 ± 1.5 at 6 months and 0.7 ± 1.0 at 12 months.

Overall, 13 patients (19.7%) experienced postoperative complications. Wound dehiscence occurred in 8 patients (12.1%). Radiographic nonunion was observed in 6 patients (9.1%), whereas osseous union was achieved in 60 patients (90.9%). The mean time to union was 11.7 ± 3.8 weeks.

Preoperative functional assessment revealed a mean AOFAS score of 40.8 ± 7.7, an FAAM‐ADL score of 46.3 ± 4.7, and an FAAM‐Sports score of 21.9 ± 7.7. Postoperatively, functional outcomes improved to a mean AOFAS score of 72.9 ± 14.6, an FAAM‐ADL score of 75.8 ± 12.4, and an FAAM‐Sports score of 60.6 ± 10.4. Demographic and clinical data are presented in Table 1.

**TABLE 1:** Demographic and clinical characteristics.

Variable	Total (*n* = 66)	Bone graft YES (*n* = 51)	Bone graft NO (*n* = 15)	*p* value
Age (years), mean ± SD	53.7 ± 14.2	54.2 ± 14.2	51.9 ± 14.6	0.576
Sex (female), *n* (%)	28 (42.4)	22 (43.1)	6 (40.0)	1.000
BMI (kg/m^2^), mean ± SD	25.7 ± 3.2	25.3 ± 3.2	27.2 ± 2.8	0.054
Rheumatoid arthritis, *n* (%)	14 (21.2)	12 (23.5)	2 (13.3)	0.495
Hypertension, *n* (%)	22 (33.3)	20 (39.2)	2 (13.3)	0.071
Diabetes mellitus, *n* (%)	12 (18.2)	9 (17.6)	3 (20.0)	1.000
Myocardial infarction, *n* (%)	4 (6.1)	3 (5.9)	1 (6.7)	1.000
Chronic kidney disease, *n* (%)	6 (9.1)	4 (7.8)	2 (13.3)	0.612
PCS‐12, mean ± SD	36.7 ± 3.4	36.5 ± 3.6	37.2 ± 2.3	0.415
MCS‐12, mean ± SD	48.1 ± 2.0	48.2 ± 2.1	47.7 ± 1.6	0.318
Operative time (min), mean ± SD	76.1 ± 24.2	75.3 ± 24.8	78.8 ± 22.9	0.510
Complications, *n* (%)	13 (19.7)	11 (21.6)	2 (13.3)	0.716
Wound dehiscence, *n* (%)	8 (12.1)	7 (13.7)	1 (6.7)	0.671
Osseous union, *n* (%)	60 (90.9)	47 (92.2)	13 (86.7)	0.612
Nonunion, *n* (%)	6 (9.1)	4 (7.8)	2 (13.3)	1.000
Time to union (weeks), mean ± SD	11.7 ± 3.8	10.8 ± 2.1	14.6 ± 6.4	0.093
Follow‐up (months), mean ± SD	14.5 ± 7.6	13.2 ± 7.2	19.4 ± 7.1	**0.005**

*Note:* The bold value (*p* = 0.005) shows a significantly longer follow‐up duration in the group without a bone graft.

### 3.2 Univariate Comparison: Bone Graft Versus No Bone Graft

Bone grafting was performed in 51 patients (77.3%), whereas 15 patients (22.7%) underwent subtalar arthrodesis without bone graft. The two groups were comparable in terms of age (54.2 ± 14.2 vs 51.9 ± 14.6 years) and sex distribution, whereas patients in the no graft group had a slightly higher BMI (27.2 ± 2.8 vs 25.3 ± 3.2 kg/m^2^), although this difference did not reach statistical significance (*p* = 0.054). Comorbidities and operative time were similarly distributed between groups, whereas follow‐up was longer in the no graft group (19.4 ± 7.1 vs 13.2 ± 7.2 months; *p* = 0.005).

Osseous union was achieved in 47 of 51 patients (92.2%) in the graft group and 13 of 15 patients (86.7%) in the no graft group (*p* = 0.612). Mean time to union tended to be shorter in the graft group (10.8 ± 2.1 vs 14.6 ± 6.4 weeks), although this difference was not statistically significant (*p* = 0.093). Postoperative complication rates were comparable between groups.

The data are summarized in Table 1.

### 3.3 Multivariate Analysis

Multivariable analyses were performed for osseous union, postoperative complications, and time to union, adjusting for age, sex, BMI, and bone graft use.

For osseous union, bone graft use was not an independent predictor of fusion (adjusted OR 1.15, 95% CI 0.11–11.56; *p* = 0.904), whereas increasing age was associated with a lower probability of union (OR 0.91 per year; *p* = 0.049, Table 2).

**TABLE 2:** Multivariate analysis for osseous union.

Variable	OR corrected (IC 95%)	*p*‐value
Age (years)	0.91 (0.83–1.00)	0.049
Sex (1 = F)	0.60 (0.09–3.83)	0.592
BMI (kg/m^2^)	0.89 (0.65–1.21)	0.445
Bone graft (1 = Yes)	1.15 (0.11–11.56)	0.904

For postoperative complications, bone graft use was also not independently associated with risk (adjusted OR 1.48, 95% CI 0.26–8.35; *p* = 0.657, Table 3).

**TABLE 3:** Multivariate analysis for time to union.

Variable	Ratio exp(*β*) (IC 95%)	% change (IC 95%)	*p*‐value
Age (years)	1.01 (1.00–1.01)	0.6% (0.2%–1.0%)	0.002
Sex (1 = F)	1.13 (0.97–1.32)	13.4% (−2.5%–31.9%)	0.103
BMI (kg/m^2^)	1.04 (1.02–1.05)	3.6% (1.9%–5.3%)	< 0.001
Bone graft (1 = Yes)	0.82 (0.64–1.04)	−18.5% (−36.1%–4.1%)	0.101

For time to union, higher age (*p* = 0.002) and BMI (*p* < 0.001) were independently associated with longer healing time, whereas bone graft use showed a nonsignificant trend toward faster union (−18.5%; *p* = 0.101, Table 4).

**TABLE 4:** Multivariate analysis for complications.

Variable	OR corrected (IC 95%)	*p*‐value
Age (years)	1.05 (0.99–1.12)	0.083
Sex (1 = F)	1.76 (0.44–7.06)	0.424
BMI (kg/m^2^)	1.13 (0.90–1.41)	0.294
Bone graft (1 = Yes)	1.48 (0.26–8.35)	0.657

### 3.4 ROC Analysis (Youden Index)

Delayed union was defined a priori as a time to union exceeding 12 weeks, based on commonly reported healing timelines for isolated subtalar arthrodesis. ROC analysis identified clinically relevant thresholds for increased risk across different outcomes. The risk of nonunion increased in patients aged 60 years or older. ROC analysis identified an age threshold of ≥ 60 years as the optimal cutoff for predicting nonunion, with an area under the curve (AUC) of 0.782, a sensitivity of 83.3%, and a specificity of 70.0%.

Similarly, the risk of delayed union (time to union > 12 weeks) increased in older patients. An age threshold of ≥ 59 years was identified as the optimal predictor of delayed union, with an AUC of 0.812.

**FIGURE 2:**
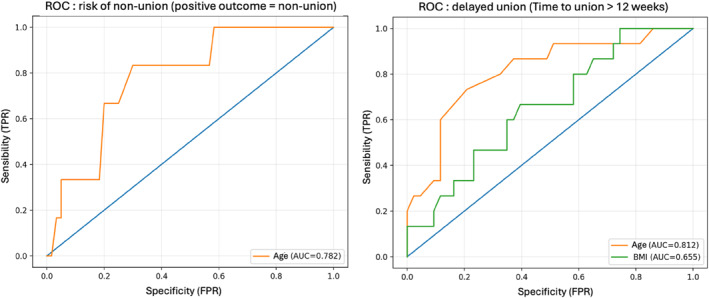
ROC curves.

In the same model, a higher body mass index was also associated with a prolonged time to union. A BMI ≥ 25.9 kg/m^2^ was identified as the optimal cutoff for predicting delayed union, with an AUC of 0.655 (Figure 2).

These findings should be interpreted as exploratory and hypothesis‐generating rather than actionable clinical thresholds.

### 3.5 Preoperative Versus Postoperative Functional Outcomes

Preoperative functional scores were comparable between the bone graft and no graft groups, with no statistically significant differences in baseline AOFAS (40.3 ± 8.1 vs 42.6 ± 6.2; *p* = 0.576) and FAAM‐ADL scores (46.4 ± 5.0 vs 46.0 ± 3.8; *p* = 0.939), whereas the preoperative FAAM‐Sports score was significantly higher in the bone graft group (24.4 ± 8.3 vs 16.9 ± 1.9; *p* < 0.001). FAAM‐Sports data were available only for a limited subset of patients, which may reflect selective completion and introduces a potential risk of nonrandom missingness. Accordingly, these results should be interpreted with caution.

Both the bone graft and no graft groups showed significant improvements in all functional scores from the preoperative to the postoperative assessment.

**FIGURE 3:**
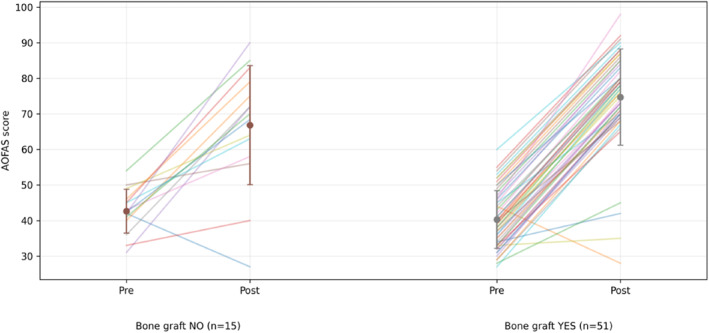
AOFAS score (preop versus postop).

**FIGURE 4:**
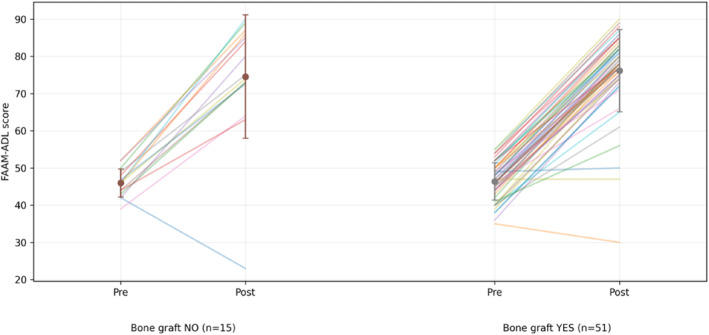
FAAM‐ADL score (preop versus postop).

**FIGURE 5:**
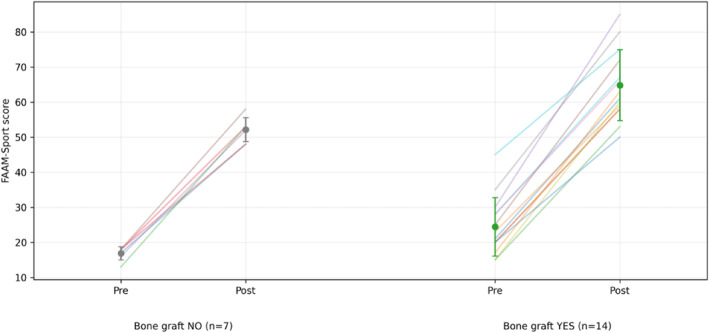
FAAM‐Sports score (preop versus postop).

In the bone graft group, the mean AOFAS score improved from 40.3 ± 8.1 preoperatively to 74.7 ± 13.5 postoperatively (*p* < 0.001), as shown in Figure 3; the FAAM‐ADL score increased from 46.4 ± 5.0 to 76.2 ± 11.0 (*p* < 0.001), as shown in Figure 4. Among patients with available FAAM‐Sports data (*n* = 14), scores improved from 24.4 ± 8.3 to 64.8 ± 10.1 (*p* < 0.001), Figure 5.

In the no graft group, the mean AOFAS score increased from 42.6 ± 6.2 to 66.8 ± 16.8 (*p* < 0.001), as shown in Figure 3; the FAAM‐ADL score increased from 46.0 ± 3.8 to 74.6 ± 16.6 (*p* < 0.001), as shown in Figure 4. FAAM‐Sports scores (*n* = 7) improved from 16.9 ± 1.9 preoperatively to 52.1 ± 3.4 postoperatively (*p* < 0.001), as shown in Figure 5.

These findings confirm a clinically meaningful functional improvement following subtalar arthrodesis in both groups, regardless of the use of bone graft.

### 3.6 Analysis According to the Bone Graft Type

Among grafted patients (*n* = 51), 24 received autologous graft (47.1%), 17 commercially allogeneic graft (33.3%), and 10 heterologous graft (19.6%).

Significant differences were observed in age (*p* = 0.028), PCS‐12 (*p* < 0.001), operative time (*p* = 0.027), follow‐up (*p* = 0.001), and AOFAS and FAAM‐ADL scores (both *p* < 0.001), as shown in Table 5.

**TABLE 5:** Graft group.

Variable	Total (*n* = 51)	Autologous (*n* = 24)	Commercial allogeneic (*n* = 17)	Fresh frozen allogeneic (*n* = 10)	*p*‐value
AGE (y)	54.16 ± 14.23	49.54 ± 13.66	55.18 ± 15.13	63.50 ± 9.23	0.028
Sex (F), *n*/*N* (%)	22/51 (43.1%)	7/24 (29.2%)	8/17 (47.1%)	7/10 (70.0%)	0.076
BMI (kg/m^2^)	25.32 ± 3.19	26.30 ± 3.39	24.69 ± 2.79	24.03 ± 2.86	0.099
RHEUMATOID ARTHRITIS	12/51 (23.5%)	4/24 (16.7%)	5/17 (29.4%)	3/10 (30.0%)	0.613
HYPERTENSION	20/51 (39.2%)	6/24 (25.0%)	8/17 (47.1%)	6/10 (60.0%)	0.159
DIABETES	9/51 (17.6%)	5/24 (20.8%)	2/17 (11.8%)	2/10 (20.0%)	0.804
MYOCARDIAL INFARCTION	3/51 (5.9%)	0/24 (0.0%)	2/17 (11.8%)	1/10 (10.0%)	0.286
CHRONIC KIDNEY DISEASE	4/51 (7.8%)	1/24 (4.2%)	2/17 (11.8%)	1/10 (10.0%)	0.823
PCS‐12	36.52 ± 3.64	39.24 ± 1.92	34.86 ± 2.96	32.83 ± 2.91	< 0.001
MCS‐12	48.22 ± 2.11	48.76 ± 1.71	47.46 ± 2.33	48.22 ± 2.41	0.152
OPERATIVE TIME (min)	75.31 ± 24.80	78.17 ± 16.40	80.65 ± 34.83	59.40 ± 14.71	0.027
COMPLICATIONS	11/51 (21.6%)	4/24 (16.7%)	3/17 (17.6%)	4/10 (40.0%)	0.342
Wound dehiscence	7/51 (13.7%)	4/24 (16.7%)	1/17 (5.9%)	2/10 (20.0%)	0.566
NON UNION	4/51 (7.8%)	0/24 (0.0%)	2/17 (11.8%)	2/10 (20.0%)	0.100
OSSEOUS UNION	47/51 (92.2%)	24/24 (100.0%)	15/17 (88.2%)	8/10 (80.0%)	0.102
TIME TO UNION (weeks)	10.83 ± 2.07	11.00 ± 2.02	10.67 ± 1.88	10.62 ± 2.72	0.852
FOLLOW‐UP (months)	13.16 ± 7.15	10.54 ± 6.47	18.88 ± 6.30	9.70 ± 3.56	0.001
AOFAS score preop	40.29 ± 8.11	45.62 ± 6.43	37.59 ± 6.54	32.10 ± 4.36	< 0.001
AOFAS score postop	74.71 ± 13.53	82.79 ± 7.07	69.88 ± 14.19	63.50 ± 13.18	< 0.001
FAAM‐ADL score preop	46.39 ± 5.02	47.04 ± 4.86	47.47 ± 4.95	43.00 ± 4.47	0.05
FAAM‐ADL score postop	76.18 ± 11.04	81.21 ± 4.69	73.47 ± 14.40	68.70 ± 10.41	< 0.001
FAAM‐Sport score preop	24.43 ± 8.31	20.57 ± 4.20	26.60 ± 7.44	32.50 ± 17.68	0.274
FAAM‐Sport score postop	64.79 ± 10.10	59.86 ± 4.41	72.60 ± 10.14	62.50 ± 17.68	0.113

Multivariable analyses were performed in the subgroup of patients in whom the type of bone graft was clearly defined (*n* = 51), including autologous graft (*n* = 24, 47.1%), commercial allogeneic graft (*n* = 17, 33.3%), and fresh frozen allogeneic graft (*n* = 10, 19.6%), with autologous graft used as the reference category. Models were adjusted for age, sex, BMI, and, for functional outcomes, the corresponding preoperative score. Direct comparisons between commercial allogeneic and fresh frozen allogeneic grafts were obtained through post‐estimation contrasts.

Regarding clinical outcomes, the graft type was not independently associated with osseous union, time to union, or postoperative complications. Both commercial allogeneic and fresh frozen grafts showed no statistically significant differences compared with autologous graft in terms of union probability, and no graft type significantly influenced the duration of bone healing after adjustment for patient‐related factors. Similarly, the risk of postoperative complications did not differ significantly among graft types.

In contrast, differences emerged when evaluating postoperative functional outcomes. In the adjusted model for FAAM‐ADL, the use of commercial allogeneic graft was independently associated with lower postoperative scores than autologous graft (*β* −8.03 points; *p* = 0.012). No statistically significant difference was observed between fresh frozen allogeneic and autologous grafts for FAAM‐ADL, nor in the direct comparison between commercial and fresh frozen allogeneic grafts (Table 6).

**TABLE 6:** Adjusted model for the FAAM‐ADL score postop.

Variable	*β* (IC 95%)	*p*‐value
Age (years)	**−0.13 (−0.28–0.02)**	**0.084**
Sex (1 = F)	**−0.86 (−4.83–6.54)**	**0.767**
BMI (kg/m^2^)	**−0.25 (−1.12–0.63)**	**0.581**
Graft type: commercial allogeneic versus. autologous	**−8.03 (−14.30–1.77)**	**0.012**
Graft type: fresh frozen allogeneic versus. autologous	**−6.95 (−16.30–2.40)**	**0.145**
Graft type: commercial allogeneic versus. fresh frozen allogeneic	**1.08 (−13.17–11.02)**	**0.861**
FAAM‐ADL preop	**1.14 (0.32–1.96)**	**0.006**

*Note:* Bold values highlight significant independent predictors in multivariable regression models. These include baseline preoperative scores (all *p* ≤ 0.006) and graft type comparisons.

For FAAM‐Sports, although no significant differences were found when comparing commercial and fresh frozen allogeneic grafts individually against autologous graft, a significant difference emerged in the direct comparison between commercial allogeneic and heterologous grafts, favoring the first one (*β* + 15.4 points; *p* < 0.001). This finding should be interpreted cautiously given the limited number of patients available for FAAM‐Sports analysis (Table 7).

**TABLE 7:** Adjusted model for the FAAM‐Sports score postop.

Variable	*β* (IC 95%)	*p*‐value
Age (years)	**−0.04 (−0.48–0.40)**	**0.855**
Sex (1 = F)	**−2.15 (−9.87–14.26)**	**0.726**
BMI (kg/m^2^)	**−0.49 (−1.22–2.19)**	**0.577**
Graft type: commercial allogeneic versus. autologous	**−5.50 (−3.85–14.86)**	**0.249**
Graft type: fresh frozen allogeneic versus. autologous	**−9.90 (−26.01–6.21)**	**0.229**
Graft type: commercial allogeneic vs. fresh frozen allogeneic	**15.40 (8.08–22.73)**	**<** **0.001**
FAAM Sport preop	**1.00 (0.62–1.38)**	**<** **0.001**

*Note:* Bold values highlight significant independent predictors in multivariable regression models. These include baseline preoperative scores (all *p* ≤ 0.006) and graft type comparisons.

No statistically significant associations between the graft type and postoperative AOFAS scores were observed after multivariable adjustment (Table 8).

**TABLE 8:** Adjusted model for the AOFAS score postop.

Variable	*β* (IC95%)	*p*‐value
Age (years)	**−0.10 (−0.38–0.18)**	**0.496**
Sex (1 = F)	**−0.68 (−7.94–6.59)**	**0.855**
BMI (kg/m^2^)	**−0.59 (−1.46–0.28)**	**0.183**
Graft type: commercial allogeneic versus. autologous	**−7.06 (−15.45–1.33)**	**0.099**
Graft type: fresh frozen allogeneic versus. autologous	**−8.67 (−18.12–0.77)**	**0.072**
Graft type: commercial allogeneic versus. fresh frozen allogeneic	**1.61 (−10.02–13.25)**	**0.786**
AOFAS preop	**0.76 (0.29–1.24)**	**0.002**

*Note:* Bold values highlight significant independent predictors in multivariable regression models. These include baseline preoperative scores (all *p* ≤ 0.006) and graft type comparisons.

Among high‐risk patients defined as patients aged > 59 years and/or with BMI > 25.9 kg/m^2^, an adjusted model was performed. Preoperative AOFAS was positively associated with postoperative AOFAS (*β* = 1.27, 95% CI 0.69–1.86; *p* < 0.001). Bone graft use was independently associated with higher postoperative AOFAS than no grafting (*β* = 15.36, 95% CI 2.72–28.01; *p* = 0.017), after adjustment for baseline AOFAS, age and BMI. Age was not significantly associated with postoperative AOFAS (*β* = −0.40 per year, 95% CI −0.92 to 0.13; *p* = 0.137), whereas BMI showed a nonsignificant trend toward lower postoperative AOFAS (*β* = −1.75 per kg/m^2^, 95% CI −3.70 to 0.19; *p* = 0.077).

## 4 Discussion

The present multicenter retrospective observational study evaluated the clinical and radiological outcomes of subtalar arthrodesis, with a specific focus on the role of bone grafting and on the comparison between graft and no graft techniques. In addition, a subgroup analysis was conducted to investigate potential differences among autologous, fresh frozen allogeneic, and commercial allogeneic bone grafts. All subgroup and threshold analyses should therefore be interpreted as exploratory and hypothesis‐generating.

An important consideration when interpreting the present findings is the heterogeneity of bone graft types included in the analysis. Autologous, fresh frozen allogeneic, and commercially available grafts differ in their biological and mechanical properties and cannot be considered equivalent. However, this heterogeneity reflects real‐world clinical practice in multicenter settings, where graft selection is often influenced by availability, surgeon preference, and patient‐specific factors rather than standardized protocols. Importantly, the present study was not designed or powered to provide definitive comparative conclusions between graft subtypes, and no formal subgroup analyses were performed due to the limited sample size within each graft category. Therefore, the findings related to the graft type should be interpreted as exploratory and hypothesis‐generating.

The use of bone grafting in subtalar arthrodesis has traditionally been advocated in selected clinical scenarios, particularly in the presence of hindfoot deformity, bone loss, or compromised bone stock. In the setting of deformity correction, bone grafting plays a key role in restoring alignment, filling osseous defects, and maintaining correction; however, residual gaps at the fusion interface may also be created by the joint preparation itself, even in the absence of overt deformity. This is particularly relevant in cases such as a cavus talus or intrinsic asymmetry of the subtalar joint, where preparation of the posterior facet may exacerbate preexisting incongruities.

Importantly, these conditions were not represented in the present cohort, as patients with hindfoot malalignment were excluded. Accordingly, the results of this study should be interpreted in the context of isolated subtalar arthrodesis performed in a well‐aligned hindfoot, where the indication for bone grafting is less clearly defined and often reflects surgeon preference rather than a strict biomechanical requirement.

Scranton et al. advocated the use of bone grafting to reduce the risk of nonunion [12], whereas Kitaoka et al. and Tasto et al. reported 100% union rates without the use of bone graft, concluding that grafting is not mandatory to achieve successful fusion [20, 21]. Similarly, Dahm et al. reported that bone grafting was not essential for achieving union in subtalar arthrodesis, although their cohort consisted of patients treated following intra‐articular calcaneal fractures [22]. Joveniaux et al. further evaluated patients undergoing subtalar arthrodesis with and without bone grafting and found no statistically significant differences in time to union between the two groups [23].

In the present series, patients treated with and without bone graft achieved comparable rates of osseous union (92.2% and 86.7%, *p* = 0.612). Godoy‐Santos et al. [24] supported the concept that in the absence of hindfoot malalignment, successful subtalar fusion primarily depends on adequate joint preparation and stable fixation rather than on the routine use of bone graft. Cardoso et al. assessed that a successful bony fusion depends on a more complex relationship of several factors other than patients' comorbidities as several technical and mechanical factors have also been related determinants for nonunion development [25]. Kim et al. highlighted the influence of biological and mechanical factors on union success, especially in posttraumatic subtalar arthrodesis cohorts [26].

In our study, although time to union tended to be shorter in the graft group (10.8 ± 2.1 vs. 14.6 ± 6.4 weeks), this difference was not statistically significant (*p* = 0.093), suggesting that any biological advantage provided by grafting, as reported by Panteli et al. [27], is limited when mechanical and surgical factors are optimal [26, 28, 29].

Consistent with this interpretation, bone graft use was not independently associated with osseous union or postoperative complications, confirming that grafting alone does not overcome unfavorable host conditions. In contrast, increasing age was associated with a reduced probability of union, and both age and BMI were independently associated with longer time to union. An age threshold of ≥ 60 years was associated with nonunion, and both aged ≥ 59 years and BMI ≥ 25.9 kg/m^2^ were associated with delayed union. Berlet et al. suggested that age is an identifiable and a concerning risk factor for hindfoot and ankle arthrodesis nonunion [30]. Overweight and obesity have been shown in multiple prior studies to increase the complication rate following elective and nonelective orthopedic surgery [31, 32, 33]. Cherukuri et al. reported that BMI has been found to be inversely related to bone mineral density, influencing both the quality and quantity of accessible autograft materials [34].

Subgroup analysis according to the graft type showed no significant differences in union rate, time to union, or complication rates among autologous, commercial allogeneic, and fresh frozen allogeneic grafts. This finding is consistent with literature indicating comparable fusion outcomes across various graft materials. A recent systematic evaluation of Bolia et al. suggested that both autografts and allografts provide adequate fusion support, and synthetic substitutes have shown outcomes similar to autograft in many scenarios [35]. Hoveidaei et al. reported that synthetic bone grafts show promise in achieving comparable outcomes to autografts in radiological, clinical, and quality‐of‐life measures, while also minimizing complications [36]. Fusco et al. argued that a novel submicron surface topography biphasic calcium phosphate offers a promising bone graft substitute for arthrodesis [37]. In his study, Gauthier et al. showed that joints treated with no biological adjuvant, allograft, or autograft demonstrated equivalent fusion rates [38]. In our cohort, although all graft types were associated with substantial functional improvement, autologous bone graft consistently demonstrated the highest postoperative functional scores, particularly for both AOFAS (45.62 ± 6.43 preop vs. 82.79 ± 7.07 postop, *p* < 0.001) and FAAM‐ADL (47.04 ± 4.86 preop vs. 81.21 ± 4.69 postop, *p* < 0.001). However, these findings should be interpreted cautiously, as baseline functional differences and residual confounding may have contributed to the observed postoperative score variations despite multivariable adjustment. Autogenous bone graft has several advantages, including histocompatibility, osteogenicity, osteoconductive and osteoinductive properties, and no risk of disease transmission [39]. On the other hand, acquiring the autograft from the patient is an additional operation posing several complications related to donor‐site bone harvesting. In our study, donor‐site pain following autologous iliac crest bone grafting was most pronounced in the early postoperative period (mean VAS 5.83 ± 1.05 at 1 month) and progressively decreased over time, reaching low residual levels at 6 and 12 months. Complications including blood loss, chronic pain at donor site, infection, nerve injury, and increased surgical duration and expenses may occur due to autograft bone harvesting [40]. Nevertheless, no donor‐site complications were observed in our cohort, except postoperative pain. Given the comparable radiographic outcomes among graft types, synthetic and heterologous grafts appear to represent valid alternatives, particularly in patients in whom donor‐site morbidity or operative time should be minimized.

## 5 Limitations and Clinical Implications

The present study has several limitations that should be acknowledged. First, its retrospective design inherently introduces the risk of selection bias, and graft choice was not randomized but based on surgeon preference and intraoperative findings. Second, although the multicenter design increases external validity, variability in surgical technique and postoperative management among centers cannot be completely excluded. Third, radiographic assessment of fusion was primarily based on standard imaging, and computed tomography was not routinely performed in all cases, potentially affecting the accuracy of union assessment. This may have influenced both the estimation of union rates and the assessment of time to union, particularly in cases of partial or delayed fusion. Computed tomography was not routinely performed due to its retrospective nature and variability in institutional follow‐up protocols, which limited the feasibility of standardized CT‐based fusion assessment.

In addition, the subgroup analysis according to the graft type was limited by the smaller sample size, particularly for functional outcome measures, which may reduce statistical power and limit definitive conclusions. Notably, patients treated with fresh frozen allogeneic bone graft were older and had lower baseline PCS‐12 scores than the other graft groups. This baseline imbalance may have introduced a selection bias and represents a potential confounding factor when interpreting the differences in outcomes among graft types. The imbalance in group sizes between graft and no graft cohorts may have introduced residual confounding despite statistical adjustment.

Furthermore, no patients in the present cohort were treated with synthetic bone grafts; therefore, the findings related to the graft type are limited to comparisons between autologous and allogeneic bone grafts and cannot be extrapolated to synthetic substitutes. Although donor‐site pain was prospectively assessed at predefined time points, other aspects of donor‐site morbidity, such as sensory disturbances, cosmetic concerns, or patient satisfaction, were not systematically evaluated and may therefore be underestimated. The absence of randomization and the limited follow‐up duration in some patients may have influenced the evaluation of long‐term outcomes. Additionally, follow‐up duration differed between the graft and no graft groups, which may have influenced the detection of delayed complications or nonunion. The use of linear regression for time to union, rather than survival analysis, represents a methodological limitation and was chosen due to the limited number of events and retrospective nature of the dataset.

Finally, the heterogeneity of graft types and the smaller sample size within each subgroup limit the ability to draw definitive conclusions regarding the comparative effectiveness of specific graft materials. Moreover, as analyses were primarily conducted by considering the graft group as a whole, any inferences drawn should be interpreted with caution, given the intrinsic heterogeneity of this group. Therefore, the findings related to the graft type should be interpreted with caution and considered hypothesis‐generating rather than confirmatory.

Despite this limitation, the chosen analytical approach was considered acceptable for exploratory purposes, as the majority of unions occurred within a narrower postoperative time window.

## 6 Conclusion

In this multicenter retrospective cohort of patients undergoing isolated SJA with a well‐aligned hindfoot, high union rates and significant functional improvement were observed irrespective of bone graft use. However, due to the nonrandomized design, surgeon‐driven graft selection, limited number of nonunion events, and exploratory nature of the analyses, these findings do not support definitive conclusions regarding the necessity or superiority of routine bone grafting. Importantly, in patients at higher risk of impaired healing, such as older individuals and those with increased body mass index, bone graft use was associated with improved functional outcomes and a trend toward shorter time to union. Although these observations should be interpreted cautiously, they suggest that bone grafting may provide a clinical advantage in selected high‐risk patients. Accordingly, bone graft selection should be individualized, taking into account patient‐specific risk factors, local bone quality, and surgeon experience.

## Author Contributions

**Giovan Giuseppe Mazzella:** conceptualization, methodology, supervision, writing – review and editing. **Antonio Bove:** project administration, data curation, investigation, writing – original draft. **Andrea De Fazio:** investigation, data curation, writing – review and editing. **Natale Maria Gangemi:** resources, investigation, validation. **Marco Peruzzi:** resources, data curation. **Fabrizio Forconi:** investigation, validation, resources. **Philip Krekosch:** investigation, data curation, formal analysis. **Christian Lausmann:** methodology, investigation, validation. **Mustafa Citak:** supervision, methodology, writing – review and editing. **Vincenzo Di Sanzo:** resources, investigation. **Giulio Maccauro:** supervision, project administration, funding acquisition. **Raffaele Vitiello:** conceptualization, methodology, supervision writing – review and editing.

## Funding

This study was supported by institutional funds of the Università Cattolica del Sacro Cuore.

## Conflicts of Interest

The authors declare no conflicts of interest.

## Data Availability

The data that support the findings of this study are available from the corresponding author upon reasonable request.
